# *In Vitro* Bioaccessibility, Human Gut Microbiota Metabolites and Hepatoprotective Potential of Chebulic Ellagitannins: A Case of Padma Hepaten^®^ Formulation

**DOI:** 10.3390/nu7105406

**Published:** 2015-10-13

**Authors:** Daniil N. Olennikov, Nina I. Kashchenko, Nadezhda K. Chirikova

**Affiliations:** 1 Laboratory of Medical and Biological Research, Institute of General and Experimental Biology, Siberian Division, Russian Academy of Science, Sakh’yanovoy Street 6, Ulan-Ude 670-047, Russia; ninkk@mail.ru; 2 Department of Biochemistry and Biotechnology, North-Eastern Federal University, 58 Belinsky Street, Yakutsk 677-027, Russian; hofnung@mail.ru

**Keywords:** Padma Hepaten, chebulic ellagitannins, bioaccessibility, human gut microbiota, urolithins, Tibetan medicine, Bras Bu, Triphala, hepatoprotective activity

## Abstract

Chebulic ellagitannins (ChET) are plant-derived polyphenols containing chebulic acid subunits, possessing a wide spectrum of biological activities that might contribute to health benefits in humans. The herbal formulation Padma Hepaten containing ChETs as the main phenolics, is used as a hepatoprotective remedy. In the present study, an *in vitro* dynamic model simulating gastrointestinal digestion, including dialysability, was applied to estimate the bioaccessibility of the main phenolics of Padma Hepaten. Results indicated that phenolic release was mainly achieved during the gastric phase (recovery 59.38%–97.04%), with a slight further release during intestinal digestion. Dialysis experiments showed that dialysable phenolics were 64.11% and 22.93%–26.05% of their native concentrations, respectively, for gallic acid/simple gallate esters and ellagitanins/ellagic acid, in contrast to 20.67% and 28.37%–55.35% for the same groups in the non-dialyzed part of the intestinal media. Investigation of human gut microbiota metabolites of Padma Hepaten and pure ChETs (chebulinic, chebulagic acids) established the formation of bioactive urolithins (A, B, C, D, M5). The fact of urolithin formation during microbial transformation from ChETs and ChET-containing plant material was revealed for the first time. Evaluation of the protective effect of ChETs colonic metabolites and urolithins on *tert*-butyl hydroperoxide (*t*-BHP)-induced oxidative injury in cultured rat primary hepatocytes demonstrated their significant reversion of the *t*-BHP-induced cell cytotoxicity, malonic dialdehyde production and lactate dehydrogenase leakage. The most potent compound was urolithin C with close values of hepatoprotection to gallic acid. The data obtained indicate that in the case of Padma Hepaten, we speculate that urolithins have the potential to play a role in the hepatic prevention against oxidative damage.

## 1. Introduction

Interest in phytochemicals has grown as their consumption has increased, and after observing their biotransformation in body tissues associated with several health benefits. After the application of phytochemicals, a series of *in vivo* biotransformations occur that can affect the bioaccessibility and bioavailability of the ingested plant compounds [1]. A large number of prospective studies have been applied to *in vitro* systems that enable phytochemical changes during gastrointestinal digestion to be predicted [2,3,4,5]. Thereby a certain body of information about processes resulting in the digestion of such groups of compounds as carotenoids, flavonoids, and phenylpropanoids has been accumulated [6,7,8,9,10]. Among the mentioned compounds, tannins are secondary metabolites of plant origin that possess very important bioactivities [11,12,13,14], but relatively little scientific information has been devoted to the investigation of biotransformations of these compounds [2,3]. In this connection, studies of the bioaccessibility and transformation processes during the digestion of tannin-containing raw material are relevant.

Tannin-containing plants are a major group of medicinal raw materials, especially in the practice of traditional medical systems. Analyzing the range of polyherbal formulations used in Tibetan medicine, one of the most frequently used combination is a three-component mixture consisting of *T. chebula*, *T. bellirica*, and *E. officinalis* and named *Three fruit* or *Bras Bu*. This combination is also well known in Ayurveda, where it is called *Triphala*. According to ethnopharmacological data the combination *Bras Bu/Triphala* is traditionally used as laxative for chronic constipation, colon cleansing, digestion problems, poor food assimilation [15,16]. It is also used as a cardioprotective, hypotonic, and an anti-ulcer remedy [16,17].

The main components of the *Bras Bu*, *T. chebula* and *T. bellirica,* are rich in ellagitannins and tannin-related compounds. A special feature of *Terminalia* species is their ability to accumulate a unique group of ellagitannins containing residues of chebulic acid in their structures (chebulanin, chebulagic acid, chebulinic acid, *etc.*) [18]. The high content of chebulic ellagitannins in *Terminalia* species and *Terminalia* containing remedies resulted in concluding they have a leading role in demonstrating biological activity [19,20,21]. Early studies on ellagitannins and ellagitannin-containing products revealed that their intake is associated with the formation of bioactive colonic metabolites urolithins [22] and possessed good bioavailability [23]. Several biological activities have been proven for urolithins, including anti-inflammatory [24,25,26,27,28,29,30,31] and antioxidant action [27,28]. In this context it is reasonable to assume that urolithins are biologically active metabolites formed in the human organism and produce the biological activity in *Terminalia*-containing formulations. Unfortunately, due to the absence of any information about the bioaccessibility and colonic transformations of chebulic ellagitannins, their direct contribution to therapeutic effects is discussed controversially.

The preparation Padma Hepaten^®^ (also Padma Liver Regulator) is the modern representation of the classical formula *Bras Bu* from Tibetan Medicine, which is produced by Padma AG in Switzerland according to international pharmaceutical quality guidelines. The formula is used for weak liver function, e.g., in the recovery after hepatitis. It is also recommended as hepatoprotector for detoxification and preventing damage of the liver [32]. An experimental study shows that the administration of Padma Hepaten significantly decreased the severity of CCl_4_ induced hepatic fibrosis and ALT serum levels [33]. Despite the available information concerning the biological activities of Padma Hepaten data about its bioaccessibility and biotransformation processes during digestion are absent. Therefore, the aim of the current study was to investigate the main chemical composition of Padma Hepaten, to determine the bioaccessibility of compounds during a gastrointestinal *in vitro* dynamic digestion model, as well as to evaluate the *ex vivo* transformation of Padma Hepaten tannins by human fecal microbiota and to estimate the hepatoprotective potentials of colonic metabolites against *tetr-*butyl hydroperoxide-induced experimental hepatocyte injury.

## 2. Experimental Section

### 2.1. Materials

Padma Hepaten capsules were provided by Padma AG (Hinwil, Switzerland). Pepsine from porcine gastric mucosa powder (7125, LOT 066K0779RD2, 600 units/mg solid) and bile extract porcine (8631) was purchased from Sigma Chemical Co. (Sigma-Aldrich Canada Ltd., Oakville, ON, Canada). Pancreatin from porcine pancreas (A0585, LOT 1G003907) was purchased from AppliChem GmbH (Darmstadt, Germany). HPLC (high performance liquid chromatography) grade acetonitrile was purchased from Cryochrom (St. Petersburg, Russia). Brain heart infusion broth as well as sodium chloride, sodium phosphate monobasic, sodium bicarbonate, potassium chloride, potassium phosphate monobasic, magnesium chloride, calcium chloride dihydrate, lithium perchlorate, ammonium chloride, sodium hydroxide, hydrochloric acid, and perchloric acid, *tetr-*butyl hydroperoxide (*t*-BHP) were purchased from Sigma-Aldrich (St. Louis, MO, USA). Reference compounds with purity greater than 95% were used. This included commercially available compounds: gallic acid, glucogallin, α/β-punicalagins, corilagin, chebulinic acid, and ellagic acid from Sigma-Aldrich (St. Louis, MO, USA); 1,6-di-*O*-galloyl-β-d-glucose, 3,4,6-tri-*O*-galloyl-β-d-glucose, chebulic acid, chebulanin, and chebulagic acid from Chelwill Asia Co., Ltd. (Beijing, China). Urolithins A, B, and C were synthesized according to Bialonska *et al.* [27]. Dialysis tubing benzoylated (D7884-10FT, 099K7007, avg. flat width 32 mm) were purchased from Sigma-Aldrich (St. Louis, MO, USA). BD GasPak™ EZ Anaerobe container system sachets were purchased from BD Company (New Jersey, NJ, USA). Minisart^®^ plus syringe filters (0.2 μm) were purchased from Sartorius Stedium Biotech GmbH (Goettingen, Germany).

### 2.2. MC-RP-HPLC-UV Conditions

#### 2.2.1. HPLC Apparatus

The HPLC analyses were performed using the microcolumn liquid chromatographic system MiliChrom A-02 (Econova, Novosibirsk, Russia) equipped with a dual low-pressure gradient pump with vacuum degasser, an autosampler, a column compartment, and a UV detector.

#### 2.2.2. HPLC Conditions for Separation of Padma Hepaten Phenolics

Separation was carried out with ProntoSIL-120-5-C18 AQ analytical column (1 mm × 60 mm × 5 μm; Metrohm AG; Herisau, Switzerland). Column temperature was maintained at 35 °C. Elution was conducted using eluent A (0.2 М LiClO_4_ in 0.006 M HClO_4_) and eluent B (acetonitrile) with a two-step gradient as follows: 0.00–4.25 min 5%–25% B, 4.25–5.00 min 25%–100% B. The flow rate was 600 μL/min. 1 μL of each sample was introduced by autosampler to the column. The column was equilibrated 4 min between injections. Chromatograms were quantified based at 254 nm. UV spectra were recorded in the range of 190–400 nm.

#### 2.2.3. Preparation of Standard Solutions of Phenolics for Quantification

Stock solutions of standards were made by accurately weighing 1 mg of gallic acid, glucogallin, α/β-punicalagins, corilagin, chebulinic acid, ellagic acid, 1,6-di-*O*-galloyl-β-d-glucose, 3,4,6-tri-*O*-galloyl-β-d-glucose, chebulic acid, chebulanin, and chebulagic acid and dissolving each in 1 mL of methanol in a volumetric flask. As all the compounds used for quantification were well-separated in experiment conditions mixtures of standards were analyzed. Prepared solutions were stored at 4 °C for no more than 72 h.

#### 2.2.4. Sample Preparation for Quantification

An accurately weighed, dried, and powdered Padma Hepaten (50 mg) was placed in a conical flask. 4 mL of 60% methanol was added and the mixture was weighted. The sample was then extracted for 30 min at 40 °C in a UZV-2.8 ultrasonic device (Sapfire, Moscow, Russia) with an ultrasound power of 100 W and frequency of 35 kHz, equipped with a temperature controller and a digital timer. After cooling, the flask weight was reduced to the initial sign, and the resultant extract was filtered through a 0.22-μm PTFE syringe filter before injection into the HPLC system for analysis.

For the preparation of the phenolic fraction from intestine media retentate, 1 mg of extract was placed in an Eppendorf tube, 1 mL of 60% ethanol was added, and the mixture was weighed. Then the sample was extracted in an ultrasonic bath for 10 min at 40 °C. After cooling, the tube weight was reduced to the initial sign, and the resulting extract was filtered through a 0.22-μm PTFE syringe filter before injection into the HPLC system for analysis.

### 2.3. Simulated Gastrointestinal Digestion in Dynamic Conditions

#### 2.3.1. Preparation of Digestive Fluids

The simulated digestive fluids were prepared fresh daily as described in the United States Pharmacopeia [34] with some modifications as follows [35,36].

*Simulated Gastric Fluid.* Aliquots of 61.0 mL NaCl (200.0 g/L), 11.7 mL NaH_2_PO_4_ (88.8 g/L), 35.8 mL KCl (89.6 g/L), 70.0 mL CaCl_2_·2H_2_O (22.2 g/L), 39.0 mL NH_4_Cl (30.6 g/L), and 32.5 mL HCl (37%) were mixed in a volumetric flask and the total volume was adjusted to 250 mL by distilled water. Then the solution was supplemented by HCl up to pH 2.0 (solution I). The simulated gastric fluid was prepared before use by mixing pepsin (400 mg) with the 25 mL of solution I (stored at 4 °C).

*Simulated Intestinal Fluid.* Aliquots of 75.0 mL NaCl (200.0 g/L), 75.0 mL NaHCO_3_ (84.7 g/L), 19.0 mL KH_2_PO_4_ (8 g/L), 12.0 mL KCl (89.6 g/L), and 19.0 mL MgCl_2_ (5 g/L) were mixed in a volumetric flask and the total volume was adjusted to 200 mL by distilled water (solution II). The simulated intestinal fluid was prepared before use by mixing pancreatin (40 mg) with 4 mL of solution II.

*Simulated Bile Solution*. The simulated bile solution consisted of bile (50 mg) dissolved in 10 mL of solution contained 2.93 mL NaCl (175.3 g/L), 6.65 mL NaHCO_3_ (84.7 g/L), 0.40 mL KCl (89.6 g/L), and 0.02 mL HCl (37%).

#### 2.3.2. Simulated Gastrointestinal Digestion

*Simulated Gastric Digestion*. Accurately weighted, dried, and powdered sample of Padma Hepaten (400 mg) was incubated with freshly prepared SGF (25 mL, pH 2.0) in a 50 mL Erlenmeyer flask for 60 min at 37 °C in a shaking water bath (167 rpm). The gastric digestion phase was terminated by inactivating pepsin by raising the pH of the solution to 7.0 with the addition of 1 M NaOH.

**Figure 1:**
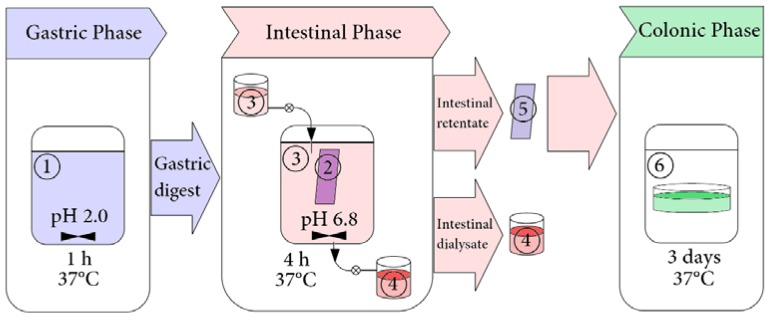
Schematic diagram of the experimental design. **①**—sample of Padma Hepaten in simulated gastric fluid; **②**—gastric phase digest in simulated intestinal fluid; **③**—simulated intestinal fluid; **④**—intestinal phase dialysate; **⑤**—intestinal phase retentate; **⑥**—anaerobic incubation of the intestinal phase retentate with fecal slurry suspension.

*Simulated Intestinal Digestion*. The dynamic model was used for estimating digestibility and was a simplified model of the two-step proteolysis model developed by Savoie and Gauthier [37]. The whole sample after gastric digestion (pH 7.0) was transferred to the dialysis bag used as the simulated small intestinal compartment. 1 mL of bile solution and 4 mL of Simulated Intestinal Fluid were added to the dialysis bag, and digestion was continued for 4 h with continuous stirring. The dialysis bag was immersed in a vessel containing buffer solution (similar in composition to Simulated Intestinal Fluid without of pancreatin addiction, 1000 mL, pH 7.0) and maintained at 37 °C while mixing. This vessel was connected to a buffer feeding reservoir (at 37 °C) and a receiving flask. The buffer solution in which the dialysis bag was immersed was constantly replenished from the feeding reservoir at a transfer rate of 1.6 mL/min using a peristaltic pump. The buffer solution with permeated digestion products was transferred to the receiving flask at the same transfer rate. The buffer solution was collected in the receiving flask at the end of intestinal phase digestion (Figure 1).

#### 2.3.3. Collection of Samples for HPLC Analysis

Samples (50 μL) were collected at 2, 4, 6, 8, 10, 20, 30, 40, 50 and 60 min of gastric digestion and at 30, 60, 90, 120, 150, 180, 210 min of intestinal digestion from retentate. Intestine media dialysate was collected at 210 min of dialysis. Plant residue after the ending of intestine phase of digestion was centrifuged and analyzed as undigested sample. HPLC samples were neutralized (if need), and then freeze-dried. Samples were filtered through 0.2 μm syringe filters before injection into the HPLC system for analysis. HPLC conditions were similar to Section 2.2.

### 2.4. Ex vivo Conditions of Microbial Metabolites Formation with Human Gut Microbiote

#### 2.4.1. Sample Preparation

We developed the techniques for preparation of extract from the retentate liquid of intestine media after the dialysis process by means of liquid-liquid extraction followed by an organic solvent treatment and a solid phase extraction on RP-SiO_2_. As an extractant for pure ellagitannins, a mixture of *n*-butanol and ethyl formate in a ratio 1:1 was used. The composition of the extractant was chosen after preliminary investigation of extraction ability of various organic solvents and their mixtures. The treatment protocol for the retentate resulting from intestinal digestion of the herbal preparation was as follows. An aliquot of retentate (300 mL) was mixed with extractant solution in a ratio 2:1 and shaken at 100 rpm for 20 min. After separation of the layers the organic layer was collected. Liquid-liquid extraction was repeated three times. Combined organic layers were concentrated *in vacuo* at 40 °C to its dry residue, dissolved in 10 mL of 90% ethanol, centrifuged at 6000 g and placed on the cartridge of RP-SiO_2_ (2 g) preconditioned with 20 mL of 90% ethanol and 30 mL of water. The cartridge was washed by 50 mL of water and 100 mL of 80% ethanol. The resulting ethanolic solution was concentrated *in vacuo* to dry residue. The yield of the phenolic fraction from intestine media retentate (PFIMR) was 45.2 ± 1.81 mg.

#### 2.4.2. Incubation of the Samples

Human fecal samples were donated by healthy volunteers aged 25 to 30 years without a history of gastrointestinal disease. Donors had not used antibiotics in the six months before sample collection. The study complied with the Helsinki Declaration. The intake of ellagitannin-containing products was strictly forbidden for one week before sample collection. Samples were processed within 30 min from defecation. The growth medium brain heart infusion (BHI) was prepared according to the manufacturer’s instructions. To acquire anaerobic conditions, BHI was boiled and immediately cooled down immediately before the experiment. Fecal slurries (FS) were prepared by suspending 1 g of human feces in 10 mL of BHI (37 °C). 40 mg of the PFIMR or 10 mg of pure compounds (chebulinic acid, chebulagic acid) were dissolved in 1 mL of distilled water and sterilized by filtration through 0.2 μm syringe filters. 1 mL of FS and 0.5 mL of PFIMR solution or of pure compounds solution were added to 8.5 ml of BHI (37 °C). 0.5 mL of distilled water was added to the control sample. The batch cultures were incubated in a sealed container under anaerobic conditions at 37 °C at 72 h (Figure 1). As the control, incubations of PFIMR or pure compounds without FS and FS without PFIMR or pure compounds were performed.

#### 2.4.3. Collection of Samples for HPLC Analysis

Samples (200 μL) were collected at the time points 0, 12, 24, 36, 48, 60 and 72 h of the incubation. An aliquot of the sample (100 μL) was placed on the cartridge of RP-SiO_2_ (0.5 g) preconditioned with 5 mL of methanol and 10 mL of water. The cartridge was washed with 5 mL of water and 1 mL of methanol. The resulting methanolic solution was filtered through a 0.2 μm syringe filters before injection into the HPLC system for analysis.

#### 2.4.4. HPLC Conditions for Separation of Human Gut Microbiota Metabolites

MC-RP-HPLC-UV-MS analyses were performed using the apparatus described in Section 2.2.1. Separation was carried out with ProntoSIL-120-5-C18 AQ analytical column (1 mm × 60 mm × 5 μm; Metrohm AG; Herisau, Switzerland). Column temperature was maintained at 40 °C. Elution was conducted using eluent A (0.4 М LiClO_4_ in 0.01 M HClO_4_) and eluent B (acetonitrile) with a two-step gradient as follows: 0–15 min 5%–100% B. The flow rate was 100 μL∙min^−1^. 1 μL of each sample was introduced to the column by autosampler. The column was equilibrated for 6 min between injections. Chromatogramms were quantified based at 310 nm. UV spectra were recorded in the range of 190–500 nm. The LC eluate was introduced into the ESI interface (LCMS-8030 Triple Quadrupole Mass Spectrometer; Shimadzu; Kyoto, Japan) without splitting and compounds were analyzed in the negative mode (nebulizer pressure of 40 psi; drying gas flow rate of 9 L/min; N_2_ temperature of 300 °C; capillary voltage of 4.5 kV; mass scan range 100–2000 *m*/*z*). For identification of compounds retention time (*t*_R_), UV-patterns and MS-data used compared with reference substances and literature data [21,27,31].

#### 2.4.5. Preparation of Standard Solutions of Ellagic Acid, Chebulinic Acid, Chebulagic Acid, and Urolithins for Quantification

Stock solutions of standards were made by accurately weighing 1 mg of ellagic acid, chebulinic acid, chebulagic acid, urolithin A, urolithin B and urolithin C and dissolving each in 1 mL of methanol–DMSO (10:1) mixture in a volumetric flask. As all the compounds used for quantification were well-separated in experiment conditions mixtures of standards were analyzed. Prepared solutions were stored at 4 °C for no more than 48 h. The contents of components were calculated from calibration curves that were constructed using ellagic acid, chebulinic acid, chebulagic acid, urolithins A, B and C. Content of urolithins M5 and D were calculated using urolithin C as a standard.

### 2.5. Ex vivo Conditions of t-BHP-Induced Oxidative Hepatotoxicity in Cultured Rat Hepatocytes

Rat hepatocytes were prepared by collagenase perfusion, as described Lee *et al.* [38]. The cell viability was determined by tryptophan blue exclusion, and was found to be greater than 95%. The cells were incubated in a humidified CO_2_-incubator CB 53 (Binder GmbH, Tuttlingen, Germany) at 37 °C in an air atmosphere. The medium was replaced with fresh medium 4 h after plating. At 20 h after plating, the media was replaced with L-15 media containing the test chemicals. The cells were either seeded onto 24-well plates at 1.8 × 10^5^ cells/well for cytotoxicity study (MTT assay and lactate dehydrogenase (LDH) leakage), or 6-well plates at 9 × 10^5^ cells∙well^−1^ for malonic aldehyde (MDA) measurement. All treatments were performed 20 h after cell attachment to allow for monolayer formation. After cell attachment, the cells were incubated in L-15 medium for 4 h with gallic acid, urolithins A, B and C (1, 5, 25 and 50 μg/mL) followed by *t*-BHP treatment (2.0 mM). For the control group neither chemicals nor *t*-BHP were added. The cell viability was measured by the MTT assay [39]. LDH leakage was determined spectrophotometricaly at 340 nm [38]. The lipid peroxidation product, MDA was measured tiobarbituric acid fluorimetric assay involving emission at 552 nm and excitation at 515 nm using 1,1,3,3-tetramethoxypropane as a standard [40].

### 2.6. Statistical Analysis

Statistical analyses were performed using a one-way analysis of variance (ANOVA), and the significance of the mean difference was determined by Duncan’s multiple range test. Differences at *p* < 0.05 were considered statistically significant. The results are presented as mean values ± SD (standard deviations) of the three replicates.

## 3. Results

### 3.1. HPLC Fingerprint of Padma Hepaten

**Figure 2:**
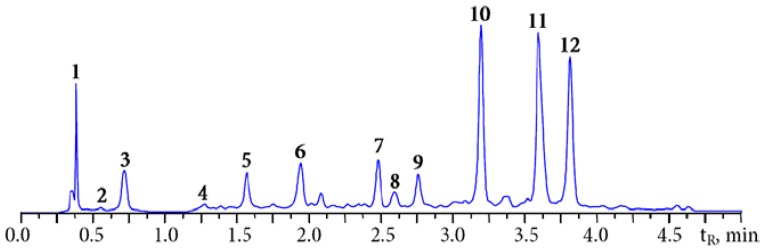
HPLC chromatogram (254 nm) of Padma Hepaten extract. Compounds: **1**—chebulic acid; **2**—glucogallin; **3**—gallic acid; **4**—1,6-di-*O*-galloyl-β-d-glucose; **5**—α-punicalagin; **6**—β-punicalagin; **7**—corilagin; **8**—3,4,6-tri-*O*-galloyl-β-d-glucose; **9**—chebulanin; **10**—chebulagic acid; **11**—ellagic acid; **12**—chebulinic acid.

In the preliminary stage of the investigation we determined the composition of the main components of Padma Hepaten сapsules using MC-RP-HPLC-UV technique previously applied for the analysis of the main phenolics in *Terminalia* and *Phyllanthus emblica* fruit [41,42]. Figure 2 presents a typical HPLC-chromatogram of a batch of Padma Hepaten сapsules. At least 12 compounds were present in detectable levels including gallic acid (**3**) and simple gallate esters—glucogallin (**2**), 1-*O*-galloyl-β-d-glucose), 1,6-di-*O*-galloyl-β-d-glucose (**4**), 3,4,6-tri-*O*-galloyl-β-d-glucose (**8**); non-chebulic ellagitannins—corilagin (**7**); 1-*O*-galloyl-3,6-*O*-(*R*)-hexahydroxydiphenoyl-β-d-glucose), α-punicalagin (**5**); 2,3-*O*-(*S*)-hexahydroxydiphenoyl-4,6-O-(*S,S*)-gallagyl-α-d-glucose), β-punicalagin (**6**); 2,3-*O*-(*S*)-hexahydroxydiphenoyl-4,6-O-(*S,S*)-gallagyl-β-d-glucose); chebulic acid (**1**) and chebulic ellagitannins – chebulanin (**9**); 1-*O*-galloyl-2,4-*O*-chebuloyl-β-d-glucose), chebulagic acid (**10)**; 1-*O*-galloyl-2,4-*O*-chebuloyl-3,6-*O*-hexahydroxydiphenoyl-β-d-glucose), chebulinic acid (**12**); 1,3,6-tri-*O*-galloyl-2,4-*O*-chebuloyl-β-d-glucose); and ellagic acid (**11**). Marker compounds of both *Terminalia* species were chebulic ellagitannins **1**, **9**, **10**, **12** and non-chebulic ellagitannins **5**–**7**. The presence of specific components of *P. emblica* fruit (**2**, **3**) was also observed in Padma Hepaten.

According to the results obtained in the quantitative analysis of the Padma Hepaten the chebulic ellagitannins were the main phenolics (185.42 mg/g of dry weight) (Table 1). The content of non-chebulic ellagitannins was 43.73 mg/g and for gallic acid and simple gallate esters it was 43.05 mg/g. The percentage of ellagic acid was *ca*. 2% (16.14 mg/g).

**Table 1:** Phenolic content in Padma Hepaten plant mixture (±SD).

Compound	Content, mg/g
*Chebulic acid and chebulic ellagitannins*	**185.42**
Chebulic acid	6.73 ± 0.18
Chebulanin	13.89 ± 0.33
Chebulagic acid	84.94 ± 1.95
Chebulinic acid	79.86 ± 1.83
*Non-chebulic ellagitannins*	**43.73**
α-Punicalagin	12.27 ± 0.31
β-Punicalagin	15.13 ± 0.36
Corilagin	16.33 ± 0.34
*Gallic acid and simple gallate esters*	**43.05**
Gallic acid	18.36 ± 0.42
Glucogallin	8.45 ± 0.18
1,6-Di-*O*-galloyl-β-d-glucose	7.17 ± 0.14
3,4,6-Tri-*O*-galloyl-β-d-glucose	9.07 ± 0.19
*Other compounds*	**16.14**
Ellagic acid	16.14 ± 0.51
*Total*	**288.36**

Chebulagic acid, chebulinic acid and α/β-punicalagins were dominant compounds and their contents were 84.94, 79.86 and 27.40 mg/g respectively. The total content of the three mentioned compounds was ~20% of the dry plant weight and constituted more than 60% of the identified chemical compound.The total content of the identified component in the batch analyzed was 288.36 mg/g. On the basis of the investigations performed it can be claimed that phenolic compounds are the major components of Padma Hepaten and are responsible for the presence of the biological activity of this supplement.

### 3.2. Release of Phenolic Compounds from Padma Hepaten During In vitro Dynamic Gastrointestinal Digestion

#### 3.2.1. Gastric Phase of Digestion

**Figure 3:**
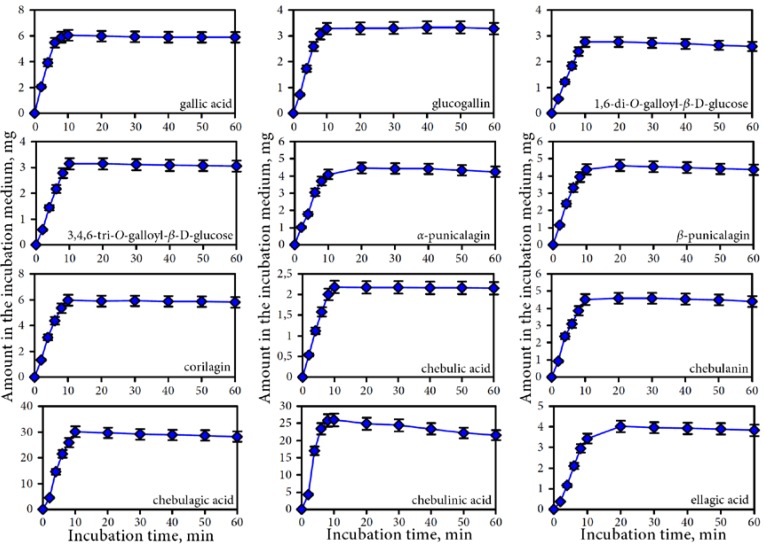
Dynamic of release of phenolic compounds in gastric media for separate compounds.

The results of a gastric phase of digestion demonstrate that the release of phenolic compounds in the gastric juice is a complex process consisting of several stages. At the beginning of the process a quick release of phenolic compounds in the gastric environment is observed (Figure 3).

The maximum concentrations of gallic acid, glucogallin, 1,6-di-*O*-galloyl-β-d-glucose, 3,4,6-tri-*O*-galloyl-β-d-glucose, corilagin, chebulic acid, chebulagic acid and chebulinic acid were reached within 10 min. For α-punicalagin, β-punicalagin, chebulanin, and ellagic acid the highest contents were observed after 20 min of incubation. During the further incubation up to 60 min the concentrations of the ellagitannins (chebulic and non-chebulic type) were reduced by 11.17% to 27.77% of the maximal concentration, with the exception of chebulinic acid, where with 32.55% the reduction was most pronounced. Gallic acid and simple gallate esters were relatively stable in the acid environment of the gastric juice during the observed period. The reduction of the concentrations of phenolic compounds at the end of gastric phase of digestion were 7.90% (gallic acid/simple gallate esters), 17.55% (non-chebulic ellagitannins), 24.12% (chebulic acid/chebulic ellagitannins), and 40.62% (ellagic acid).

The recovery at this stage of digestion for individual groups of compounds were 92.10% for gallic acid and simple gallate esters, 82.45% for non-chebulic ellagitannins, 75.88% for chebulic acid and chebulic ellagitannins, and 59.38% for ellagic acid. The most extractable compounds were glucogallin (recovery 97.04%), gallic acid (recovery 95.00%), and 1,6-di-*O*-galloyl-β-d-glucose (recovery 90.24%) (Table 2). The lowest recovery rates were observed for ellagic acid (recovery 59.38%) and chebulinic acid (recovery 67.45%).

**Figure 4:**
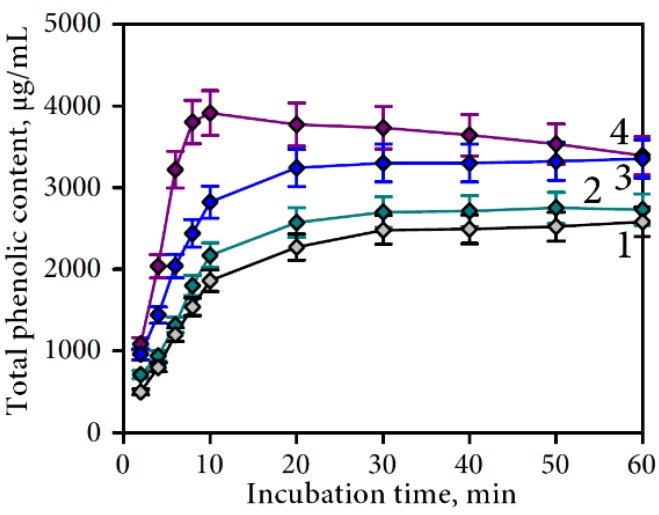
Effects of extraction media on the total phenolic compounds yield. Media: **1**—water; **2**—0.2% NaCl/water, pH 6.8, **3**—0.2% NaCl/water, pH 2.0 (HCl); **4**—pepsin (400 U/mL)/0.2% NaCl/water, pH 2.0 (HCl).

To determine the influence of the main gastric juice components (NaCl, HCl, pepsin) on the release of phenolics from Padma Hepaten phytomatrix we additionally investigated extraction potentials of four fluids: water (control medium No. 1), 0.2% NaCl solution with pH 6.8 (NaCl-containing medium No. 2), 0.2% NaCl solution with pH 2.0 (NaCl/HCl-containing medium No. 3), and 0.2% NaCl/pepsin solution with pH 2.0 (NaCl/pepsin/HCl-containing medium No. 4). The results for the aqueous extract (medium No. 1) showed that the yield increased up to 30 min of extraction (2.48 mg/mL) (Figure 4) but did not increase in longer extraction. 0.2% NaCl extraction (medium No. 2) is characterized by the same yield to extraction time curve (in 30 min point—2.7 mg/mL). More pronounced changes were observed in the case of the HCl-supplemented 0.2% NaCl (medium No. 3).

**Table 2:** Amount and recovery of phenolic compounds in undigested sample, gastric, and intestine media and plant residue obtained following *in vitro* gastrointestinal digestion of Padma Hepaten.

Compound	Undigested Sample		Gastric Media		Intestine Media Retentate		Intestine Media Dialysate		Plant Residue after Intestine Phase		Total Recovery, %	Degradation Percentage, %
	Amount, mg	Recovery, %	Amount, mg	Recovery, %	Amount, mg	Recovery, %	Amount, mg	Recovery, %	Amount, mg	Recovery, %		
*Chebulic acid and chebulic ellagitannins*												
Chebulic acid	2.69 ± 0.07	100	2.15 ± 0.10	79.93	0.78 ± 0.04	28.99	1.21 ± 0.05	44.98	0.09 ± 0.00	3.35	77.23	22.77
Chebulanin	5.56 ± 0.15	100	4.40 ± 0.22	79.14	1.11 ± 0.05	19.96	3.07 ± 0.12	55.21	0.30 ± 0.01	5.40	80.63	19.37
Chebulagic acid	33.97 ± 0.88	100	28.18 ± 1.35	82.96	18.67 ± 0.86	54.96	7.14 ± 0.36	21.02	0.19 ± 0.00	0.56	76.55	23.45
Chebulinic acid	31.95 ± 0.87	100	21.55 ± 1.01	67.45	13.36 ± 0.65	41.82	5.80 ± 0.26	18.15	0.91 ± 0.04	2.85	62.81	37.19
*Subtotal*	**74.17**	**100**	**56.28**	**75.88**	**33.92**	**45.73**	**17.22**	**23.22**	**1.49**	**2.01**	**70.96**	**29.04**
*Non-chebulic ellagitannins*												
α-Punicalagin	4.91 ± 0.14	100	4.25 ± 0.18	86.56	3.47 ± 0.15	70.67	0.56 ± 0.02	11.40	0.04 ± 0.00	0.81	82.87	17.13
β-Punicalagin	6.05 ± 0.18	100	4.37 ± 0.19	72.23	3.20 ± 0.16	52.89	1.05 ± 0.05	17.36	n.d.	0.00	70.25	29.75
Corilagin	6.53 ± 0.19	100	5.80 ± 0.26	88.83	3.01 ± 0.16	46.09	2.40 ± 0.11	36.75	0.30 ± 0.01	4.59	87.50	12.50
*Subtotal*	**17.49**	**100**	**14.42**	**82.45**	**9.68**	**55.35**	**4.01**	**22.93**	**0.34**	**1.94**	**80.29**	**19.71**
*Gallic acid and simple gallate esters*												
Gallic acid	6.19 ± 0.14	100	5.88 ± 0.26	95.00	1.14 ± 0.06	18.42	4.72 ± 0.22	76.25	0.23 ± 0.00	3.72	82.89	17.11
Glucogallin	3.38 ± 0.08	100	3.28 ± 0.16	97.04	1.12 ± 0.05	33.14	2.14 ± 0.08	63.31	n.d.	0.00	96.39	3.61
1,6-Di-*O*-galloyl-β-d-glucose	2.87 ± 0.06	100	2.59 ± 0.13	90.24	0.56 ± 0.03	19.51	2.00 ± 0.08	69.69	n.d.	0.00	89.36	10.64
3,4,6-Tri-*O*-galloyl-β-d-glucose	3.63 ± 0.10	100	3.05 ± 0.13	84.02	0.74 ± 0.04	20.39	2.18 ± 0.09	60.06	0.13 ± 0.00	3.58	83.99	16.01
*Subtotal*	**16.07**	**100**	**14.80**	**92.10**	**3.56**	**22.15**	**11.04**	**68.70**	**0.36**	**2.24**	**86.85**	**13.15**
*Other compounds*												
Ellagic acid	6.45 ± 0.22	100	3.83 ± 0.17	59.38	1.83 ± 0.10	28.37	1.68 ± 0.07	26.05	0.66 ± 0.03	10.23	64.67	35.33
*Subtotal*	**6.45**	**100**	**3.83**	**59.38**	**1.83**	**28.37**	**1.68**	**26.05**	**0.66**	**10.23**	**64.67**	**35.33**
Total	**114.18**	**100**	**89.33**	**78.24**	**48.99**	**42.91**	**33.95**	**29.73**	**2.85**	**2.48**	**74.39**	**25.61**

n.d.: not detected.

The maximal yield was at 20 min, suggesting that the presence of HCl accelerated the extraction of phenolics from plant material. Moreover, the content of phenolics after 30 min of incubation in media was 3.30 mg/mL. Extraction with NaCl/pepsin/HCl-containing medium (medium No. 4) demonstrated that the extraction process consisted of two stages. In the first 10 min of extraction the phenolic yield increased rapidly while in second prolonged stage (10–60 min) decreasing yields of phenolics in extract were observed. Total phenolics content in extract after 10 min point was 3.88 mg/mL. After 60 min of incubation the yield was *ca.* 12% lower (3.40 mg/mL). The results indicate the existence of two competitive processes—extraction and binding of phenolics.

The investigation of the individual compounds (**1**–**12**) showed that NaCl and HCl during the entire period of incubation (60 min) had no pronounced effect on the stability of the compounds (data not shown). Hydrolysis or other destructive effects of acidic or NaCl-containing extraction medium were not observed.

However, the presence of pepsin in the extraction medium influenced the concentration of the individual phenolic compounds. So, after the incubation of chebulinic acid in NaCl/HCl/pepsin medium only a reduction of the peak area was observed without an accumulation of degradation products, suggesting the binding but not degrading properties of pepsin. In a series of experiments we determined that 1 mg of pepsin may bind 380–400 μg of chebulinic acid (this value was strongly dispersed because of the unstoichiometric character of the reaction). The determination of the binding of other phenolic components of Padma Hepaten extract showed maximal binding properties for chebulinic acid.

Gallic acid, glucogallin and ellagic acid were less actively bound (<50 μg·mg^−1^). The data about the binding activity of pepsin of individual phenolic compounds are in good agreement with the results obtained previously during the analysis of the dynamics of extraction of individual compounds in gastric media.

#### 3.2.2. Intestine Phase of Digestion

**Figure 5:**
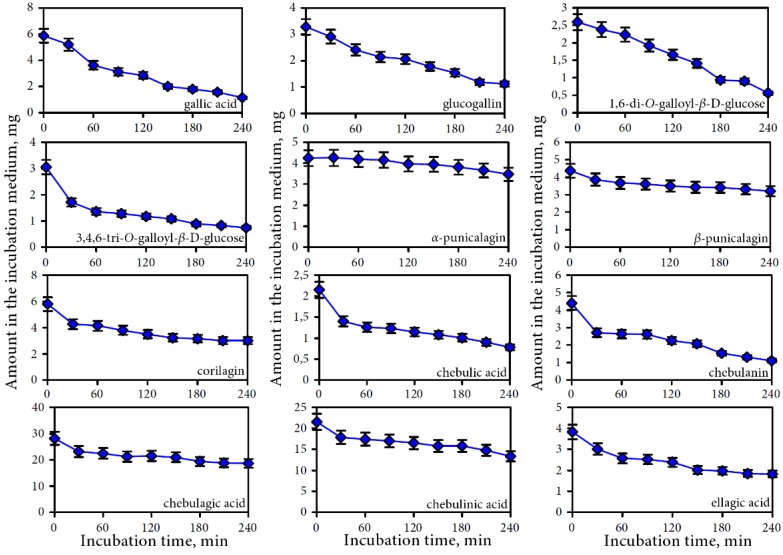
Dynamic of phenolic depletion of intestine media retentate during dialysis for separate compounds.

The intestine phase of digestion is characterized by a high pH of the digestive juices (6.8–7.5), the presence of bile acids, lipase, amylase, protease, and the ability of the transmembrane transfer for individual compounds. All of these conditions were followed in the implementation of *in vitro* digestion in dynamic experiments with semi-permeable membrane. The investigation of the dynamics of the transition process of phenolic compounds through a semi-permeable membrane showed a gradual decrease of all analyzed groups of compounds in the retentate. The rate of release on the dialysate differed for the analyzed compounds. Gallic acid and simple gallate esters were the most dyalizable compounds (Figure 5). Within 4 h, the decrease of content of gallic acid was about 80% in the retentate. The content of 1,6-di-*O*-galloyl-β-d-glucose, glucogallin and chebulanin in the retentate decreased by 78.4%, 65.8%, and 74.8%, respectively. The following compounds had the lowest rates of transition: α-punicalagin (18.4%), β-punicalagin (26.8%), chebulagic acid (33.7%), and chebulinic acid (38.0%). Gallic acid and simple gallate esters passed better through the semi-permeable membrane compared to other compounds. The values of recovery in the retentate were 22.15% for gallic acid/simple gallate esters, 28.37% for ellagic acid, 45.73% for chebulic acid/chebulic ellagitannins, and 55.35% for non-chebulic ellagitannins.

The determination of the composition of the dialysate was of particular interest, because it is regarded as the most biologically active fraction obtained in the course of the experiment. According to HPLC data intestine media dialysate is characterized by significantly higher contents of gallic acid and simple gallate esters. The value of recovery for this group of compounds was 68.70% of that in the untreated sample (Table 2).

For other compounds, these values ranged from 11.40% (α-punicalagin) to 55.21% (chebulanin). Individual groups of compounds may be arranged in descending order of their content in the dialysate as follows (% of content group from total phenolic content in intestine media dialysate): chebulic acid/chebulic ellagitannins (50.7%) > gallic acid/simple gallate esters (32.5%) > non-chebulic ellagitannins (11.8%) > ellagic acid (5.0%). For intestine media retentate this sequence is different: chebulic acid/chebulic ellagitannins (69.2%) > non-chebulic ellagitannins (19.8%) > gallic acid/simple gallate esters (7.3%) > ellagic acid (3.7%).

Analysis of the raw residue after termination of the intestinal phase of digestion showed that approximately 2.85% of phenolic compounds were not subjected to extraction. Thus, the efficiency of extraction of phenolic compounds by gastric and intestinal media was greater than 97%. Ellagic acid (recovery 10.23%), chebulanin (recovery 5.40%), and corilagin (recovery 4.59%) were the components with the lowest extraction capacities. The values of recovery of other compounds in the plant residue ranged from 0.00% to 3.72%. Final recovery rates as a sum of the recoveries in intestinal media (retentate and dialysate) and in the plant residue were 86.85% (gallic acid/simple gallate esters), 80.29% (non-chebulic ellagitannins), 70.96% (chebulic ellagitannins), and 64.67% (ellagic acid). Calculation of the difference between recovery in undigested sample and total recovery allowed us to determine the parameter of percentage degradation for the analyzed compounds. As a group the most degradable compound was ellagic acid, with 35.33% lost, followed by chebulic ellagitannins (29.04%), non-chebulic ellagitannins (19.17%), and gallic acid derivatives (13.15%).

### 3.3. Human Gut Microbiota Metabolites of Intestine Media Retentate of Padma Hepaten

**Figure 6:**
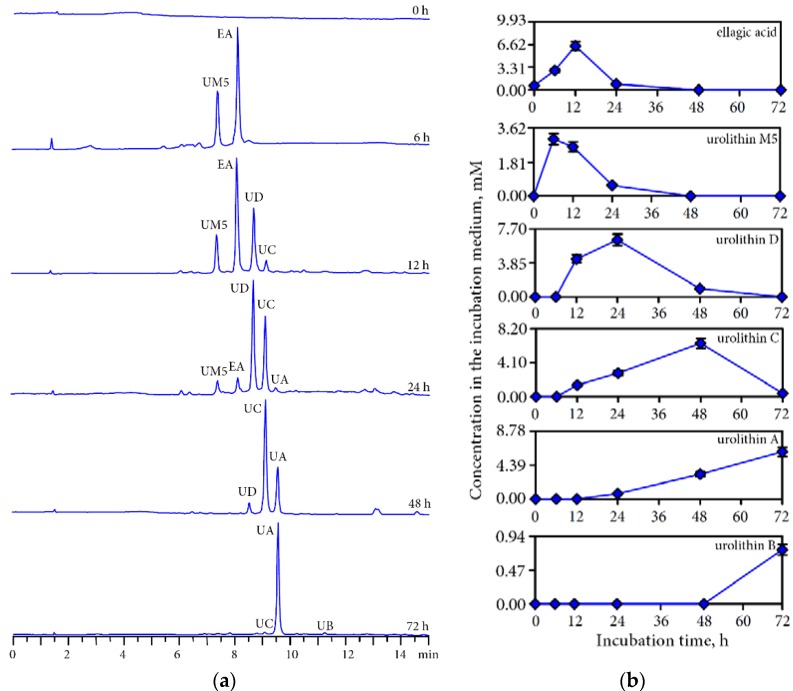
(**a**) HPLC-chromatograms of intestine media retentate after 0–72 h incubation with human gut microbiota; (**b**) Dynamic of content of ellagic acid and urolithins in colonic media.

Transformation by the action of human gut microbiota is the next stage influencing the bioavailability of phenolic compounds in the human organism. HPLC analysis of human gut microbiota *ex vivo* cultures revealed the formation of various urolithins from the phenolic fraction of Padma Hepaten intestinal media retentate. Peaks representing urolithins were identified based on their retention times and UV, MS data compared with reference compounds and literature data. The gradual change in the urolithin profile took place during 78 h of incubation of the retentate sample. At least five metabolites were detected in colonic media including ellagic acid, urolithin M5 (3,4,8,9,10-pentahydroxy-6*H*-dibenzo[*b*,*d*]pyran-6-one), urolithin D (3,4,8,9-tetrahydroxy-6*H*-dibenzo[*b*,*d*]pyran-6-one), urolithin C (3,8,9-trihydroxy-6*H*-dibenzo[*b*,*d*]pyran-6-one), urolithin A (3,8-dihydroxy-6*H*-dibenzo[*b*,*d*]pyran-6-one), and urolithin B (8-hydroxy-6*H*-dibenzo[*b*,*d*]pyran-6-one) (Figure 6a).

The first metabolites were observed when the intestinal media retentate was incubated with fecal microbiota were ellagic acid and urolithin M-5, which peaked at 12 h and 6 h, respectively, with maximum concentrations of 6.46 and 3.04 mM (Figure 6b). Then, urolithin D began reaching a maximum of 6.50 mM at 24 h. At that moment a small amount of urolithin C started to be produced, achieving a maximum of 6.43 mM at 48 h. Urolithin A was detected after 24 h of incubation, and a maximum of 6.10 mM was reached at 72 h. Only traces of urolithin B were detected in incubation media after 72 h incubation (0.90 mM). At the end of the incubation period, only urolithin A was dominantly present as well as two minor peaks of urolithins B and C.

**Figure 7:**
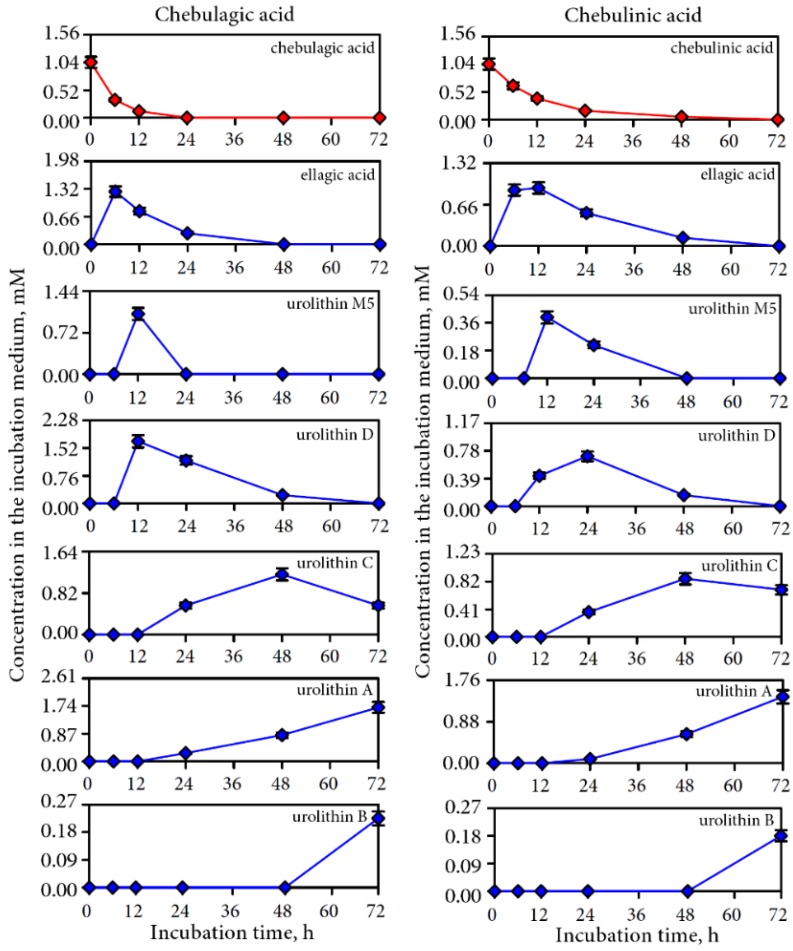
Content of chebulagic acid and chebulinic acid and their microbial metabolites in colonic media after 0–72 h incubation of pure compounds with human gut microbiota.

Urolithin production of a similar sequence was also observed for two pure ellagitannins chebulagic and chebulinic acids, the dominant compounds of the intestine media retentate, when incubated with human gut microbiota. Ellagic acid, urolithins M5, D, C, A, and B formed in colonic media successively (Figure 7). Although, in general, the manner of degradation was similar for both compounds it should be noted that the rate of transformation was different. While of chebulagic acid only traces were detectable after 24 h in colonic media chebulinic acid was detectable up to 48 h. The maximal content of urolithin D was reached after 12 h incubation of chebulagic acid and after 24 h in chebulinic acid.

### 3.4. Hepatoprotective Potential of Urolithins Against t-BHP-Induced Oxidative Injury of Hepatocytes

**Figure 8:**
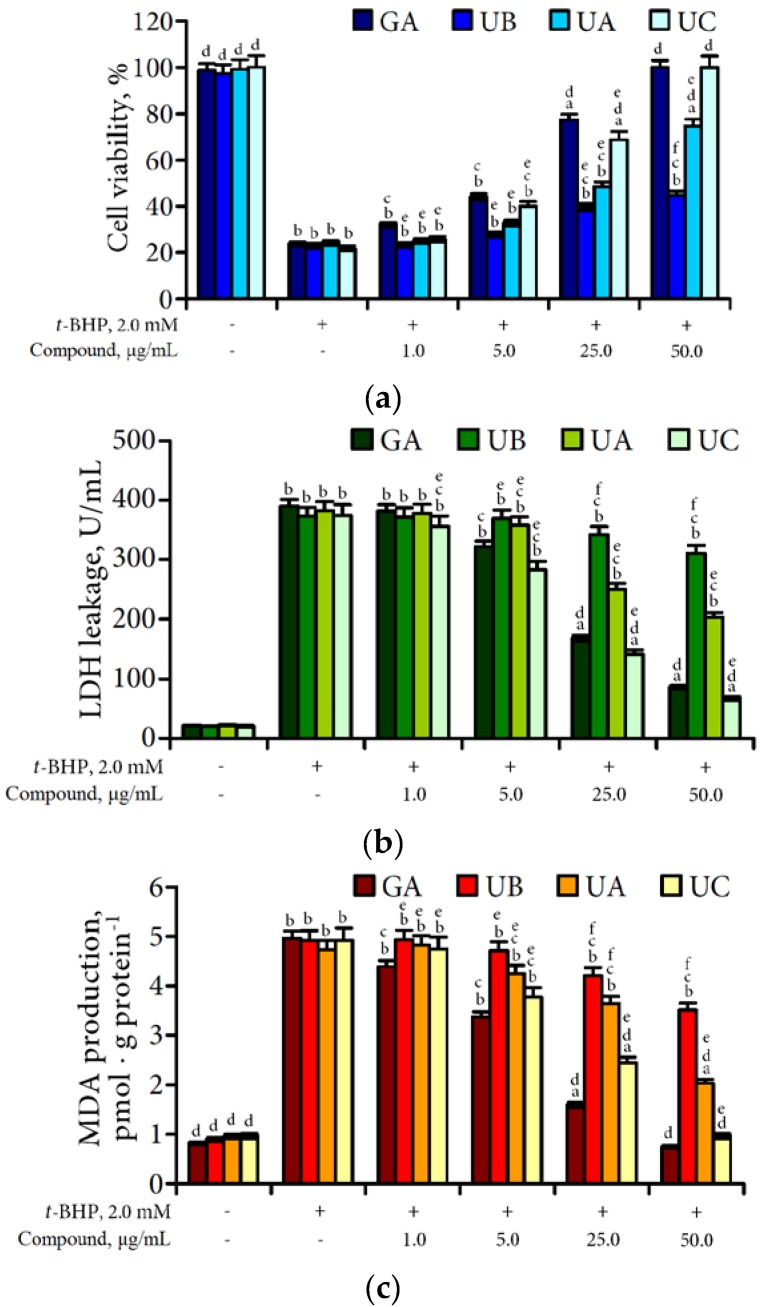
Effect of different concentrations of gallic acid (GA), urolithin A (UA), urolithin B (UB) and urolithin C (UC) on *t*-BHP-induced cytotoxicity (**a**); LDH leakage (**b**) and MDA production (**c**) in cultured rat hepatocytes. Data presented as a mean ± SD (*n* = 4). Letters indicate a significant difference: a—from the control group, *p* < 0.05; b—from the control group, *p* < 0.01; c—from the *t*-BHP-treated group, *p* < 0.05; d—from the *t*-BHP-treated group, *p* < 0.01; e—from the gallic acid treated group, *p* < 0.05; f—from the gallic acid treated group, *p* < 0.01.

An experimental model of oxidative damage of cultured rat hepatocytes by *t*-BHP was used for evaluation of hepatoprotective potential of urolithins. In the present study, the treatment of primary rat hepatocytes with *t*-BHP at concentration 2.0 mM can produce a significant decrease in cell viability of up to 21.7%–24.3% compared with control (Figure 8).

Malonic dialdehyde (MDA) production in *t*-BHP-treated cells was 4.73–4.96 pmol/g protein (*vs*. 0.82–0.97 pmol/g protein in control) and lactate dehydrogenase (LDH) leakage was 373.1–389.6 U/mL (*vs*. 20.4–22.3 U/mL in control). Observed parameters proved the strong oxidative damage of hepatocytes by *t*-BHP.

To evaluate hepatoprotective potential of chebulic ellagitannins, microbial metabolites, and urolithins against oxidative stress induced by *t*-BHP the effect of an end microbial transformation products, urolithins A, B, and C, were determined in the concentration range of 1–50 μg/mL. As a reference compound, gallic acid was chosen. Previously, gallic acid was concluded as a hepatoprotective principle of the fruits of *T. belirica* in *in vivo* bioassay guided experiments [43].

The treatment of cells with different concentrations of urolithins in the presence of *t*-BHP protected the hepatocytes against the cytotoxicity in a dose-dependent manner. At the dose 50 μg/mL of urolithin C and gallic acid, the *t*-BHP-treated cells showed fully recovered cell viability from 21.7% to 24.3% in cells treated with *t*-BHP. Viability of cells treated with gallic acid in dose 25 μg/mL (77.5%) was more than in the urolithin C group in same dose (68.8%). Application of urolithins A and B in the maximal dose (50 μg/mL) demonstrated their less effectiveness for cells protection (74.8% and 44.6%, respectively).

In case of *t*-BHP-induced LDH leakage, urolithin C demonstrated significant protective effect most pronounced in dose 50 μg/mL (67.2 U/mL *vs.* 374.3 U/mL in *t*-BHP-treated group). The reference compound, gallc acid, was less effective showing LDH activity value at 86.9 U/mL in the same dose. Despite the reduced activity of urolithins A and B, they can decrease LDH release from damaged cells in high concentrations, especially urolithin A which reduced the negative influence of *t*-BHP in maximal dose at 46.9% compared with *t*-BHP-treated group.

The general pattern of urolithin protection in case of MDA production by damaged hepatocytes was close to previously described. As shown in Figure 7, pre-treatment of *t*-BHP-induced cells with different concentration of urolithin C can significantly reduce the concentration of MDA in damaged cells almost at the initial value (50 μg/mL; 0.97 pmol/g protein *vs.* 4.92 pmol/g protein in control). Gallic acid as a known antioxidant demonstrated high effectiveness in inhibition of MDA production (50 μg/mL; 0.75 pmol/g protein *vs.* 4.96 pmol/g protein in control). Urolithin A reduced MDA production at 57.1% which, compared with the control and activity of urolithin B, was minimal (50 μg/mL; 3.51 pmol/g protein *vs.* 4.92 pmol/g protein in control).

## 4. Discussion

### 4.1. Bioaccessibility of Compounds of Padma Hepaten

This study focused on determining the bioaccessibility of the main phenolics in the three-component plant supplement Padma Hepaten, as well as on the determining the microbial transformation of ellagitannins in an *ex vivo* human gut microbiota model. Padma Hepaten is a good source of different classes of phenolic compounds including gallic acid and simple gallate esters, ellagic acid, non-chebulic ellagitannins, and chebulic ellagitannins. The latter are the dominant phenolics and amounted to 185.42 mg/g in the plant powder. The results presented in this work demonstrate that the gastric phase of Padma Hepaten digestion may be regarded as a multi-step process including at least two stages. The first stage is a rapid release of phenolics into the gastric juice with the duration of this phase at about 8–10 min. The quickness of this process may be explained by the special features of the extraction medium containing hydrochloric acid and pepsin. Our research showed that the acidic medium facilitated the yield of extractives from the plant matrix as well as the presence of pepsin influenced the extraction process by decomposing plant proteins. The second stage of digestion is a slower and longer phase characterized by a negative influence on the phenolic content of the plant extract. In this stage a gradual decrease of the content of ellagitannins (both groups chebulic and non-chebulic) was observed but not of gallic acid and its derivatives. The reason for the decrease is the formation of ellagitannin-protein complexes. The total recovery of the phenolic compounds at the end of the gastric phase of digestion ranged from 59.38% (ellagic acid) to 97.04% (glucogallin). The low recovery rate of ellagic acid may be due to its poor solubility in hydrophilic media.

After the gastric phase the dynamic intestine model with a semipermeable membrane was the next stage of digestion. The different release rates of phenolics from intestine media in dialysate fraction was observed for the different components. The gallic acid and its derivatives as well as chebulanin were the most dialyzable components. After the intestinal phase of digestion, a much lower content of phenolic compounds was present in the dialysate, the bioaccessible fraction (recovery value for total phenolics 29.44%) than in the untreated (initial) sample. A decrease of phenolics in the dialysate fraction was previously observed by many authors [2,3,9,44]. However, these investigations primarily related to studying the bioaccessibility of flavonoids, phenylpropanoids, anthocyans, catechins, and procyanidins but not tannins [2,3]. Thus, it can be claimed that the general features of changes in the chemical composition of tannin-containing formulations are close to those of other classes of phenolic compounds.

Studying the distribution of the individual compounds in the system’s retentate-dialysate revealed that gallic acid and its glucosylated analogues were the most dialyzable compounds. The bioaccessible values of four gallic acid derivatives ranged from 60.06% to 69.89%, which is almost three times more than those for chebulic ellagitannins and ellagic acid (22.93%–26.05%). These results agree well with earlier data addressing the bioavailability of gallic acid in humans, it was revealed that this compound is extremely well-absorbed compared with other polyphenols [45,46,47]. There are no scientific data, either *in vitro* or *in vivo*, concerning the bioaccessibility or bioavailability of galloylated glucoses in human. Nevertheless, its similarities in certain physico-chemical properties to gallic acid and its analogues, including high hydrophilicity and small molecule size, allow us to expect similar behavior in biological processes. In contrast to gallic acid, all tannin groups (chebulic and non-chebulic) as well as ellagic acid demonstrated a lower effectiveness in transmembrane transport. The bioaccessibility values of these groups varied from 11.40% (α-punicalagin) to 55.21% (chebulanin). The ability of tannins to bind proteins, which are obligate components of gastrointestinal liquids, is assumed to be the main reason of their decreased content in the intestine retentate. Early studies on the bioavailability of ellagitannins and ellagic acid following ingestion by humans showed no intact ellagitannins and the low concentration of ellagic acid in body tissues outside of the gastrointestinal tract [21,23,48,49,50].

### 4.2. Gut Microbiota Metabolites of Compounds of Padma Hepaten

Although *Terminalia* and *Phyllantus* species as contained in Padma Hepaten are good sources of chebulic ellagitannins, the amount of compounds available to absorption after gastrointestinal digestion is smaller. This does not mean that the undigested part in the gut content has no possible role in health protection. Non-absorbed ellagitannins can reach the large intestines where they can be transformed or degraded by the gut microflora into other biologically active metabolites like urolithins [31]. Our study reports for the first time the occurrence of urolithins in colonic media after incubation of an extract of chebulic ellagitannins with human gut microbiota. The process of microbial degradation of the investigated extract started from the enrichment of colonic media by ellagic acid followed by subsequent degradation to a series of urolithins (M5, D, C, A, B). The component profiles of urolithins in the colonic medium during the incubation were characterized in a time-dependent manner. The composition of the metabolites varied at different incubation times from penta-hydroxylated (urolithin M5) up to mono-hydroxylated analogs (urolithin B).

**Figure 9:**
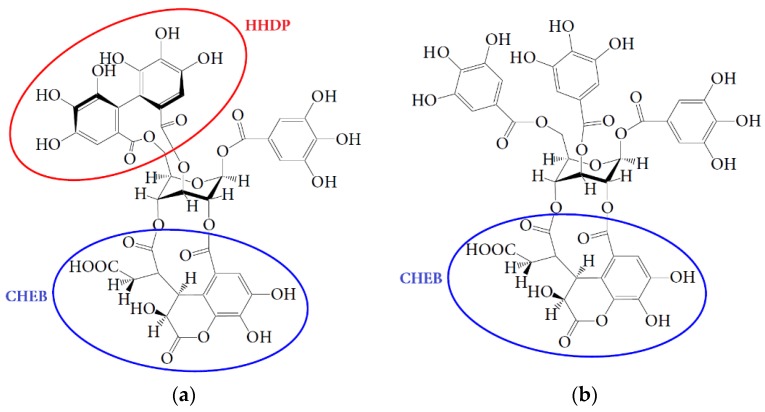
Structures of chebulagic acid (**a**) and chebulinic acid (**b**) (according [18]). Circles are framed hexahydrodiphenoyl (HHDP) and chebuloyl (CHEB) moieties.

The investigation of the microbial transformation of two dominant representatives of chebulic ellagitannins, chebulagic acid and chebulinic acid, showed a similar manner of degradation. This fact indicated that both compounds play a lead role in enrichment of colonic media by urolithins. Considering the structural features of chebulagic acid and chebulinic acid, the presence of one important difference between the compounds can be noted. The presence of a hexahydroxydiphenoyl residue (HHDP) linked at C-4 and C-6 with a fragment of β-d-glucose is typical for chebulagic acid (Figure 9).

The residues of gallic acid are attached to the C-4 and C-6 atoms of β-d-glucose for chebulinic acid. According to early conceptions the formation of ellagic acid as a precursor of urolithins in the microbial transformation of ellagitannins requires a HHDP residue [22]. In this regard, the formation of urolithins from chebulinic acid is not possible. However, as a result of our studies, the possibility of forming urolithins from chebulinic acid was presented, which indicated the presence of additional structural features that allowed microbial conversions to be achieved.

**Figure 10:**
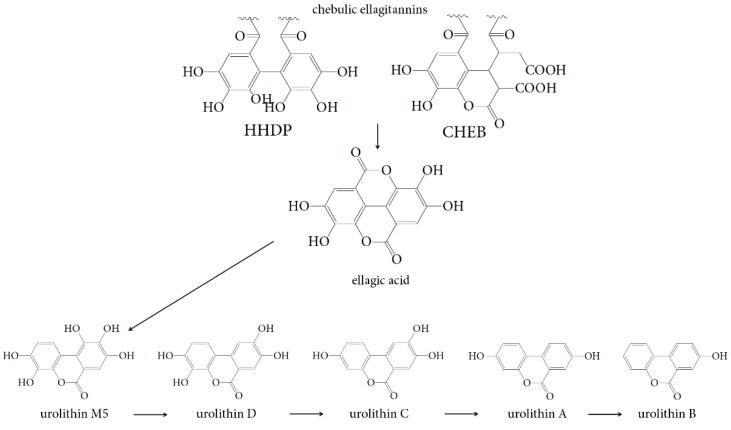
Proposed pathway of chebulic ellagitannins transformation by intestinal bacteria. HHDP—hexahydrodiphenoyl moiety, CHEB—chebuloyl moiety.

It is known that chebulinic acid, with three independent and freely rotating galloyl groups, is characterized as a more molecular flexible structure compared to chebulagic acid with its constrained HHDP moiety [18]. Taking into account this easy mobility of the galloyl groups in chebulinic acid, it can be assumed that the two residues of gallic acid at C-4 and C-6 locations have the opportunity for convergence and further conversion in fragment HHDP, from which ellagic acid is formed. Nevertheless, the involvement of a chebulinic acid fragment structurally related to ellagic acid is more probable in microbial formation of ellagic acid. According to the data on the quantitative content of transformation products of the chebulic ellagitannins in colonic medium the concentration of released ellagic acid was higher with chebulagic acid (1.26 mM), than with chebulinic acid (0.89–0.93 mM). A similar pattern was observed for urolithins. This phenomenon is probably the consequence of the fact that ellagic acid can be formed from two fragments of chebulagic acid—HHDP fragments and chebulic moiety. Thus the obtained data support the previously proposed mechanism of ellagitannins and ellagic acid transformation by intestinal bacteria (Figure 10) [22].

### 4.3. Hepatoprotective Effect of Urolithins Against t-BHP-Induced Oxidative Injury of Hepatocytes

According to the data on urolithin metabolism in mammals they are transported from the intestine to the liver, where they are transformed into glucuronides, which reach the blood and are excreted in the urine [22]. Tentatively, during this process of hepatic transformation, urolithins may show positive effects on a damaged liver structure. However, there is no scientific information demonstrating effectiveness of urolithins as hepatoprotectors. To estimate hepatoprotective potential of urolithins A, B and C as the end products of the microbial gut transformation of chebulic ellagitannins we chose the widely applied experimental model of oxidative injury of cultured rat primary hepatocytes by *t*-BHP [51].

The results of *ex vivo* experiments showed high effectiveness of urolithin C influenced positively on the viability of hepatocytes treated with a damaging agent, *t*-BHP. The fact of the significant reduction of LDH levels after application of urolithin C mean that the compound prevented the formation of surface blebs in the hepatocyte plasma membrane and does not allow to release the cytosolic enzymes in outer medium. The value of MDA production in urolithin C treated cells was lower than in urolithin A and B groups demonstrating its good antioxidant properties. An effectiveness of urolithins A and B was lower wherein urolithin B was the least active microbial metabolite. The parameters of hepatoprotection of reference compound, gallic acid, were maximal and in some cases close to the same parameters of urolithin C. In general, we can conclude that the urolithins can protect hepatocytes from the cytotoxic effects caused by damage agent *t*-BHP by preventing the oxidative injury of cells acting as antioxidants. It is known that the structure of antioxidants is the main factor of their care role on the living organisms. The antioxidant potency of the compounds has been attributed to multiple phenolic hydroxyl groups in 6*H*-dibenzo[*b*,*d*]pyran-6-one core of urolithins with potential to form *o*- or *p*-quinones. In our research, the protective effect of urolithins correlated with the number of hydroxyl groups with a higher value for urolithin C having one *o*-di-hydroxy- and one mono-hydroxy-substituted phenolic rings and lower value for urolithin B having only one mono-hydroxy-substituted phenolic ring. These data are in good agreement with the results of the previous studies used 2,2-diphenyl-1-picrylhydrazyl radical (DPPH), 2,2'-azino-bis(3-ethylbenzothiazoline-6-sulphonic acid) cation radical (ABTS^+^) [27], ferric reducing antioxidant potential (FRAP), and oxygen radical absorbance capacity (ORAC) assays [52]. Considering that ellagitannins (chebulic and non-chebulic groups) have a common mechanism of biotransformation by the colonic microbiota, it would be natural to assume that the ellagitannin containing remedies could be potential hepatoprotectors.

Based on the data obtained during the present studies, we propose the following interpretation of hepatoprotective effectiveness of Padma Hepaten. This formulation is a source of two groups of phenolic compounds with high bioaccessible values such as gallic acid and its derivatives and with low bioaccessibility as ellagitannins (both chebulic and non-chebulic groups) and ellagic acid. Both groups characterized by different ways of digestion of human organism. An uptake of gallic acid from the gastrointestinal tract into the inner media of organism started at 1–4 h after taking the medicine. Ellagic acid derivatives cannot transfer to target organ in the native form by any mechanisms that is why they undergo structural changes caused formation of urolithins. The duration of this phase may reach two to four days. Despite the different rates of assimilation of gallic acid and urolithins as a result of a periodic application of Padma Hepaten a constant uptake of bioactive compounds to the target organ should be supposed. These data support the results of an *in vivo* study in a liver fibrosis mouse model, where Padma Hepaten significantly attenuated hepatic fibrosis.

## 5. Conclusions

In this study, we investigated the componential profile of the herbal formula Padma Hepaten which is rich of chebulic ellagitannins as well as non-chebulic ellagitannins, gallic acid, simple gallate esters, and ellagic acid. Investigated compounds characterized by the various rates of bioaccessibility in the simulated gastrointestinal digestion experiments with the maximal values for the gallic acid and simple gallate esters and the lowest values for chebulic ellagitannins. Despite this fact, our data firstly showed that the chebulic ellagitannins transformed into bioaccessible colonic metabolites urolithins by gut microbiota. The high potential of urolithins to defend the hepatocytes against *t*-BHP-induced oxidative injury demonstrate the benefits of application of the herbal formula Padma Hepaten as a perspective hepatoprotective preparation.

## References

[1] Heaney R.P. (2001). Factors influencing the measurement of bioavailability, taking calcium as a model. J. Nutr..

[2] Carbonell-Capella J.M., Buniowska M., Barba F.J., Esteve M.J., Frígola A. (2014). Analytical methods for determining bioavailability and bioaccessibility of bioactive compounds from fruits and vegetables: A review. Comp. Rev. Food Sci. Food Saf..

[3] Alminger M., Aura A.M., Bohn T., Dufour C., El S.N., Gomes A., Karakaya S., Martínez-Cuesta M.C., McDougall G.J., Requena T. (2014). *In vitro* models for studying secondary plant metabolite digestion and bioaccessibility. Compr. Rev. Food Sci. Food Saf..

[4] Chen J., Gaikwad V., Holmes M., Murray B., Povey M., Wang Y., Zhang Y. (2011). Development of a simple model device for *in vitro* gastric digestion investigation. Food Funct..

[5] McClements D.J., Li Y. (2010). Review of *in vitro* digestion models for rapid screening of emulsion-based systems. Food Funct..

[6] Courraud J., Berger J., Cristol J.P., Avallone S. (2013). Stability and bioaccessibility of different forms of carotenoids and vitamin A during *in vitro* digestion. Food Chem..

[7] Garret D.A., Faila M.L., Sarama R.J. (2000). Estimation of carotenoids bioavailability from fresh stir-fried vegetables using an *in vitro* digestion/Caco-2 cell culture model. J. Nutr. Biochem..

[8] Stanisavljević N., Samardžić J., Janković T., Šavikin K., Mojsin M., Topalović V., Stevanović M. (2015). Antioxidant and antiproliferative activity of chokeberry juice phenolics during *in vitro* simulated digestion in the presence of food matrix. Food Chem..

[9] Bouayed J., Hoffmann L., Bohn T. (2011). Total phenolics, flavonoids, anthocyanins and antioxidant activity following simulated gastro-intestinal digestion and dialysis of apple varieties: Bioaccessibility and potentional uptake. Food Chem..

[10] Kern S.M., Bennett R.N., Needs P.W., Mellon F.A., Kroon P.A., Garcia-Conesa M.T. (2003). Characterization of metabolites of hydroxycinnamates in the *in vitro* model of human small intestinal epithelium Caco-2 cells. J. Agric. Food Chem..

[11] Saleem A., Husheem M., Härkönen P., Pihlaya K. (2002). Inhibition of cancer cell growth by crude extract and the phenolics of *Terminalia chebula* retz. fruit. J. Ethnopharmacol..

[12] Cho H., Jung H., Lee H., Yi H.C., Kwak H.-K., Hwang K.I. (2015). Chemopreventive activity of ellagitannins and their derivatives from black raspberry seeds on HT-29 colon cancer cells. Food Funct..

[13] Yamagata K., Tagami M., Yamori Y. (2015). Dietary polyphenols regulate endothelial function and prevent cardiovascular disease. Nutrition.

[14] Li A.N., Li S., Zhang Y.J., Xu X.R., Chen Y.M., Li H.B. (2014). Resources and biological activities of natural polyphenols. Nutrients.

[15] Baliga M.S., Meera S., Mathai B., Rai M.P., Pawar V., Palatty P.L. (2012). Scientific validation of the ethnomedicinal properties of the Ayurvedic drug Triphala: A review. Chin. J. Integr. Med..

[16] Bhatnagar S., Rani A., Kumari R. (2015). Therapeutic potential of Triphala against human diseases. Int. J. Pharm. Sci. Rev. Res..

[17] Balasubramani S.P., Venkatasubramanian P., Kukkupuni S.K., Patwardhan B. (2011). Plant-based Rasayana drugs from Ayurveda. Chin. J. Integr. Med..

[18] Pfundstein B., el Desouky S.K., Hull W.E., Haubner R., Erben G., Owen R.W. (2010). Polyphenolic compounds in the fruits of Egyptian medicinal plants (*Terminalia bellerica*, *Terminalia chebula* and *Terminalia horrida*): Characterization, quantitation and determination of antioxidant capacities. Phytochemistry.

[19] Rathinamoorthy R., Thilagavathi G. (2014). *Terminalia chebula*—Review on pharmacological and biochemical studies. Int. J. PharmTech Res..

[20] Saraswathi Motamarri N., Karthikeyan M., Kannan M., Rajasekar S. (2012). *Terminalia belerica* Roxb.—A phytopharmacological review. Int. J. Res. Pharm. Biomed. Sci..

[21] Espín J.C., González-Barrio R., Cerdá B., López-Bote C., Rey A.I., Tomás-Barberán F.A. (2007). Iberian pig as a model to clarify obscure points in the bioavailability and metabolism of ellagitannins in humans. J. Agric. Food Chem..

[22] Espín J.C., Larrosa M., García-Conesa M.T., Tomás-Barberán F. (2013). Biological significance of urolithins, the gut microbial ellagic acid-derived metabolites: The evidence so far. Evid. Based Complement. Altern. Med..

[23] Seeram N.P., Henning S.M., Zhang Y., Suchard M., Li Z., Heber D. (2006). Pomegranate juice ellagitannin metabolites are present in human plasma and some persist in urine for up to 48 h. J. Nutr..

[24] Dell’agli M., Galli G.V., Bulgari M., Basilico N., Romeo S., Bhattacharya D., Taramelli D., Bosisio M. (2010). Ellagitannins of the fruit rind of the pomegranate (*Punica granatum*) antagonize *in vitro* the host inflammatory response mechanism involved in the onset of malaria. Malar. J..

[25] Gimenez-Bastida J.A., Gonzalez-Sarrias A., Larrosa M., Tomás-Barberán F., Espín J.C., Garcia-Conesa M.T. (2012). Ellagitannin metabolites, urolithin A glucuronide and its aglycone urolithin A, ameliorate TNF-alpha-induced inflammation and associated molecular markers in human aortic endothelial cells. Mol. Nutr. Food Res..

[26] Gimenez-Bastida J.A., Larrosa M., Gonzalez-Sarrias A., Tomás-Barberán F., Espín J.C., Garcia-Conesa M.T. (2012). Intestinal ellagitannin metabolites ameliorate cytokine-induced inflammation and associated molecular markers in human colon fibroblasts. J. Agric. Food Chem..

[27] Bialonska D., Kasimsetty S.G., Khan S.I., Ferreira D. (2009). Urolithins, intestinal microbial metabolites of pomegranate ellagitannins, exhibit potent antioxidant activity in a cell-based assay. J. Agric. Food Chem..

[28] Verzelloni E., Pellacani C., Tagliazucchi D., Tagliaferri S., Calani L., Costa L.G., Brighenti F., Borges G., Crozier A., Conte A. (2011). Antiglycative and neuroprotective activity of colon-derived polyphenol catabolites. Mol. Nutr. Food Res..

[29] Gonzalez-Sarrias A., Larrosa M., Tomas-Barberan F.A., Dolara P., Espin J.C. (2010). NF-kappaB-dependent anti-inflammatory activity of urolithins, gut microbiota ellagic acid-derived metabolites, in human colonic fibroblasts. Br. J. Nutr..

[30] Kiss A.K., Granica S., Stolarczyk M., Melzig M.F. (2012). Epigenetic modulation of mechanisms involved in inflammation: Influence of selected polyphenolic substances on histone acetylation state. Food Chem..

[31] Piwowarski J.P., Granica S., Zwierzyńska M., Stefańska J., Schopohl P., Melzig M.F., Kiss A.K. (2014). Role of human gut microbiota metabolism in the anti-inflammatory effect of traditionally used ellagitannin-rich plant material. J. Ethnopharmacol..

[32] PADMA: Tibetische Arzneimittel Seit 1969: PADMA Hepaten. http://www.padma.at/produkte/padma-hepaten.

[33] Ginsburg I., Koren E., Horani A., Mahamid M., Doron S., Muhanna N., Amer J., Safadi R. (2009). Amelioration of hepatic fibrosis via Padma Hepaten is associated with altered natural killer T lymphocytes. Clin. Exp. Immun..

[34] United States Pharmacopeia (1995). Simulated Gastric Fluid and Simulated Intestinal Fluid. The United States Pharmacopeia 23, the National Formulary 18.

[35] Marambe H.K., Shand P.J., Wanasundara J.P.D. (2011). Release of angiotensin I-converting enzyme inhibitory peptides from flaxseed (*Linum usitatissimum* L.) protein under simulated gastrointestinal digestion. J. Agric. Food Chem..

[36] Oomen A.G., Rompelberg C.J.M., Bruil M.A., Dobbe C.J.G., Pereboom D.P.K.H., Sips A.J.A.M. (2003). Development of an *in vitro* digestion model for estimating the bioaccessibility of soil contaminants. Arch. Environ. Contam. Toxicol..

[37] Savoie L., Gauthier S.F. (1986). Dialysis cell for the *in vitro* measurement of the protein digestibility. J. Food Sci..

[38] Lee H.S., Won N.H., Kim K.H., Lee H., Jun W., Lee K.W. (2005). Antioxidant effects of aqueous extract of *Terminalia chebula in vivo* and *in vitro*. Biol. Pharm. Bull..

[39] Tseng T.H., Wang C.J., Kao E.S., Chu H.Y. (1996). Hibiscus protocatechuic acid protects against oxidative damage induced by *tert*-butylhydroperoxide in rat primary hepatocytes. Chem. Biol. Interact..

[40] Yagi K. (1987). Lipid peroxides and human diseases. Chem. Phys. Lipids.

[41] Olennikov D.N., Kashchenko N.I., Schwabl H., Vennos C., Loepfe C. (2015). New gallates of mucic acid from *Phyllanthus emblica*. Chem. Nat. Compd..

[42] Olennikov D.N., Kashchenko N.I., Schwabl H., Vennos C. (2015). Application of MC-RP-HPLC-UV for the rapid and sensitive analysis of phenolic compounds in *Terminalia* species. Pharm. Chem. J..

[43] Anand K.K., Singh B., Saxena A.K., Chandan B.K., Gupta V.N., Bhardwaj V. (1997). 3,4,5-Trihydroxy benzoic acid (gallic acid), the hepatoprotective principle in the fruits of *Terminalia*
*belerica*-bioassay guided activity. Pharmacol. Res..

[44] Bouayed J., Deußer H., Hoffmann L., Bohn T. (2012). Bioaccessible and dialysable polyphenols in selected apple varieties following *in vitro* digestion *vs.* their native patterns. Food Chem..

[45] Manach C., Scalbert A., Morand C., Rémésy C., Jiménez L. (2004). Polyphenols: Food sources and bioavailability. Am. J. Clin. Nutr..

[46] Shahrzad S., Bitsch I. (1998). Determination of gallic acid and its metabolites in human plasma and urine by high-performance liquid chromatography. J. Chrom. B.

[47] Shahrzad S., Aoyagi K., Winter A., Koyama A., Bitsch I. (2001). Pharmacokinetics of gallic acid and its relative bioavailability from tea in healthy humans. J. Nutr..

[48] Mertens-Talcott S.U., Jilma-Stohlawetz P., Rios J., Hingorani L., Derendorf H. (2006). Absorption, metabolism, and antioxidant effects of pomegranate (*Punica granatum* L.) polyphenols after ingestion of a standardized extract in healthy human volunteers. J. Agric. Food Chem..

[49] Crozier A., Jaganath I.B., Clifford M.N. (2009). Dietary phenolics: Chemistry, bioavailability and effects on health. Nat. Prod. Rep..

[50] Lei F., Xing D.M., Xiang L., Zhao Y.N., Wang W., Zhang L.J., Du L.J. (2003). Pharmacokinetic study of ellagic acid in rat after oral administration of pomegranate leaf extract. J. Chrom. B.

[51] Haidara K., Morel I., Abaléa V., Barré M.G., Denizeau F. (2002). Mechanism of tert-butylhydroperoxide induced apoptosis in rat hepatocytes: Involvement of mitochondria and endoplasmic reticulum. Biochim. Biophys. Acta.

[52] Pfundstein B., Haubner R., Würtele G., Gehres N., Ulrich C.M., Owen R.W. (2014). Pilot walnut intervention study of urolithin bioavailability in human volunteers. J. Agric. Food Chem..

